# Green Co-Extractant-Assisted Supercritical CO_2_ Extraction of Xanthones from Mangosteen Pericarp Using Tricaprylin and Tricaprin Mixtures

**DOI:** 10.3390/foods14172983

**Published:** 2025-08-26

**Authors:** Hua Liu, Johnson Stanslas, Jiaoyan Ren, Norhidayah binti Suleiman, Gun Hean Chong

**Affiliations:** 1 Engineering Technology Research Center, Guangzhou College of Technology and Business, Guangzhou 510850, China; annyliu@gzgs.edu.cn; 2 Faculty of Food Science and Technology, Universiti Putra Malaysia, Serdang 43400, Selangor, Malaysia; 3 Faculty of Medicine and Health Sciences, Universiti Putra Malaysia, Serdang 43400, Selangor, Malaysia; 4 Food Science and Engineering, South China University of Technology, Guangzhou 510641, China; 5 Supercritical Fluid Centre (SFC), Faculty of Food Science and Technology, Universiti Putra Malaysia, Serdang 43400, Selangor, Malaysia

**Keywords:** xanthone, mangosteen pericarp, supercritical carbon dioxide, tricaprylin, tricaprin, green co-extractant

## Abstract

Xanthones from mangosteen pericarp (MP) are bioactive compounds with promising pharmaceutical and nutraceutical applications. However, their efficient and selective extraction using environmentally friendly solvents remains a challenge. This study aimed to evaluate tricaprylin (C8) and tricaprin (C10) as novel green co-extractants in supercritical carbon dioxide (scCO_2_) extraction for the recovery of xanthones from MP, using a mass ratio of C8:C10 = 0.64:0.36, hereafter referred to as C8/C10, and to model extraction kinetics for process design and scale-up. Extraction performance was investigated using different C8/C10–MP mass ratios and scCO_2_ conditions at temperatures of 60 °C and 70 °C and pressures of 250 bar, 350 bar, and 450 bar. A pseudo-first-order kinetic model was applied to describe the extraction profile, and the kinetic parameters were generalized using second-order polynomial functions of temperature and pressure. The highest xanthone yield (39.93 ± 0.37%) and total xanthone content (51.44 ± 2.22 mg/g) were obtained at a 40% C8/C10–MP ratio under 70 °C and 350 bar, where the C8/C10 mixture outperformed other tested co-extractants in both efficiency and selectivity, particularly for α-mangostin. The extraction profiles were well described by the pseudo-first-order kinetic model, and the generalized model predicted the extraction yield with an uncertainty of 2.3%. C8/C10 is a highly effective and scalable co-extractant for scCO_2_ extraction of xanthones, offering a foundation for industrial applications in food, nutraceutical, and pharmaceutical sectors.

## 1. Introduction

Mangosteen (*Garcinia mangostana* Linn.) consists of an edible white aril and a purplish pericarp, the latter comprising approximately 75% of the total fruit weight and often discarded as agricultural waste [1]. The mangosteen pericarp (MP) is a rich source of xanthone compounds, including α-mangostin, β-mangostin, gartanin, and γ-mangostin [2,3]. Xanthones have demonstrated diverse pharmacological activities, such as antidiabetic, anti-obesity, analgesic, anti-inflammatory, neuroprotective, anti-Alzheimer, and anticancer effects [4,5,6,7,8,9]. Among these, α-mangostin is the most extensively studied, exhibiting potent antioxidant, anti-inflammatory, anticancer, antibacterial, antifungal, and wound-healing properties [10,11,12,13].

Conventional extraction of xanthones is typically performed using solvent-based techniques with ethanol, methanol, or ethyl acetate. Among these, 70% ethanol has been reported to be particularly effective for xanthone extraction from MP [14,15]. However, organic solvent extraction presents limitations such as solvent residue, long processing times, and relatively low selectivity and efficiency. In contrast, supercritical carbon dioxide (scCO_2_) extraction has emerged as a green and sustainable technology for recovering bioactive compounds [16,17]. While scCO_2_ is highly effective for extracting non-polar, lipophilic compounds, its efficiency for polar or higher molecular weight compounds is limited. To overcome this limitation, co-extractants (entrainers) are often introduced to improve solubility and mass transfer [18]. However, most reported co-extractants are organic solvents, which may lead to contamination residues from addition. Therefore, the development of green, efficient, and biocompatible co-extractants, especially from natural sources, remains a key challenge in advancing scCO_2_-based extraction processes.

Recent studies have investigated the use of edible oils as co-extractants in scCO_2_ systems to improve the recovery of plant-derived bioactives. For instance, combining scCO_2_ with virgin coconut oil (VCO), corn oil, or ghee significantly improves flavonoid extraction from propolis [19]. Similarly, the use of scCO_2_ with VCO for xanthone recovery from MP demonstrated improved efficiency and selectivity without requiring organic solvents [20,21,22]. While co-extractants can improve solubility and mass transfer, they may complicate downstream separation, particularly when high-purity isolates are required. Nonetheless, in food and nutraceutical applications, the bioactive–oil mixture can often be directly incorporated into final products without separation. Notably, Lee et al. (2019) reported that the use of VCO as a co-extractant enhanced the bioaccessibility of xanthones by up to 90% [22].

In our previous work, the solubility of xanthone was evaluated in tricaprylin (C8), tricaprin (C10), and their binary mixture. The results showed that the C8/C10 mixture exhibited better solubility than either pure component or VCO [23]. The maximum solubility was achieved at a C10:C8 mass ratio of 0.36:0.64. Therefore, this study aims to evaluate the effectiveness of a binary mixture of C8 and C10 (C8:C10 = 0.64:0.36, *w*/*w*) as a green co-extractant system in scCO_2_ extraction of xanthones from MP. The extraction performance was assessed under various co-extractant loadings, temperatures (60 °C and 70 °C), and pressures (250 bar, 350 bar, and 450 bar). A pseudo-first-order kinetic model was applied to characterize the extraction profiles. The resulting kinetic parameters, namely the extraction rate constant and maximum yield, were subsequently generalized as functions of pressure and temperature using second-order polynomial equations. These generalized models offer predictive capability for process design and optimization, highlighting the potential of the C8:C10 system as a scalable and efficient co-extractant for xanthone recovery.

## 2. Materials and Methods

### 2.1. Materials

Xanthones were procured from McLean Chemical Technology Co., Ltd. (Shanghai, China). Virgin coconut oil (VCO), tricaprylin (C8), and tricaprin (C10) were obtained from Huaxiang Kejie Biotechnology Co., Ltd. (HeFei, China). All solvents used in this study were of analytical grade, and double-distilled water, with conductivity < 0.5 μS/cm, was obtained from a water purification system. The chemical structures and details of xanthone, C8, and C10 are presented in Table S1, and fatty acid profiling of virgin coconut oil is presented in Table S2. Food-grade carbon dioxide (CO_2_, 99.9%) was supplied by Essen Biotechnology Co., Ltd. (Newark, NJ, USA). All reagents used for HPLC analysis, including phosphoric acid (H_3_PO_4_, 85.0%, general agent), acetonitrile (CH_3_CN, 99.9%, PanReac Applichem, Darmstadt, Germany), methanol (CH_3_OH, 99.9%, PanReac Applichem, Darmstadt, Germany), ethanol (C_2_H_5_OH, 99.9%, Merck, Darmstadt, Germany), and deionized water (H_2_O), were of HPLC grade.

### 2.2. Supercritical Carbon Dioxide Extraction

MP was cleaned with water and dried in a hot air oven (DR-H205G, Derui, Dongguan, China) at 60 °C for 4 h. The final water content of MP was determined with a moisture analyzer (Q1-100A, Mide, Xiamen, China) and reported as 6.1% (wet basis). The dried MP was then ground with a grinder (BF-500, Ben Chen, Shijiazhuang, China) to obtain MP powder. The powder was sieved through a series of mesh sieves, and particles within the size range of (30–300) μm with a median size of 120 μm were selected for this study.

**Figure 1:**
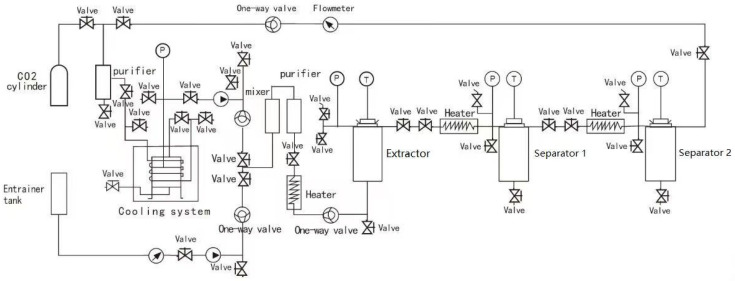
Schematic diagram of supercritical fluid extraction.

The schematic diagram of the scCO_2_ extraction setup used in this study is presented in Figure 1. A mixture of tricaprylin (C8) and tricaprin (C10) in a 0.64:0.36 ratio, referred to as C8/C10, was used as the co-extractant, using scCO_2_ as a solvent. A total of 180 g of MP was homogeneously mixed with C8/C10 at ratios of 20%, 30%, and 40% (*w*/*w*) and loaded into a filter bag. Based on preliminary experiments, the maximum co-extractant loading was limited to 40% (*w*/*w*), as higher oil content caused sample clumping, which hindered CO_2_ penetration and negatively affected extraction efficiency. The prepared filter bag was placed inside the extraction vessel (1 L; dimensions: 350 mm × 60 mm). The experiments were performed at various pressures (250 bar, 350 bar, and 450 bar) and temperatures (60 °C and 70 °C) with a CO_2_ flow of (1.02 ± 0.20) kg/h. Mangosteen pericarp extract (MPE) was collected from separator 1 and separator 2 at regular intervals and weighed. The total extraction time of 240 min was fixed based on the observation that the extract yield reached a plateau. The overall extraction curve (OEC) was constructed by plotting the cumulative yield of MPE against time.

The collected MPE samples were purged with nitrogen and sealed in airtight containers and stored in the dark at −18 °C until further analysis. All experiments were performed in triplicate. For comparative purposes, extractions were also conducted using pure scCO_2_, as well as scCO_2_ combined with individual co-extractants or co-solvents, including VCO, C8, C10, and ethanol.

The extraction yield was calculated from Equation (1), written as follows [21]:

(1) $$Extraction\;yield,\%\left(w/w\right)\;=\;\frac{mass\;of\;MPE\;\times \;100}{mass\;of\;MP+mass\;of\;co-extractant}\;\%$$

### 2.3. Determination of Xanthones in Mangosteen Pericarp Extract

In this study, a high-performance liquid chromatography system (e2695 and 2695, Waters, Milford, MA, USA) was used with modifications based on a previously reported method [24,25]. To prepare the test solution, an appropriate volume of the liquid sample was mixed with acetonitrile to dissolve and dilute the sample to a final concentration of approximately 1.0 mg/mL. Chromatographic analysis was performed using a Waters Symmetry C18 column (4.6 mm × 250 mm, 5 µm particle size), Milford, MA, USA. A total of 0.1% phosphoric acid aqueous solution was used as mobile phase A, and acetonitrile-methanol (1:1, *v*/*v*) was used as mobile phase B; the flow rate was set to 1.0 mL/min. Gradient elution was performed under the following procedures: (0–10) min, 30% A/70% B; (10–55) min, 100% B; (56–68) min, 30% A/70% B. Detection was performed at 240 nm [26,27]. The wavelength was chosen based on the absorption characteristics of the analytes to optimize sensitivity and specificity.

The chromatographic system’s performance was validated by confirming the elution order of eight xanthones based on their retention times: isomangostin, garcinone D, garcinone C, 8-deoxygartanin, γ-mangostin, gartanin, α-mangostin, and β-mangostin. Retention times for each compound were consistent across replicates, ensuring reproducibility. The content of each compound in the MPE was quantified using the external standard method, with calibration curves established for the respective standard compounds. Calibration curves were prepared with at least five concentration levels and demonstrated linearity (R^2^ > 0.99) across the tested range. The concentrations of the eight xanthones in MPE were calculated based on these calibration curves, as shown in Figure S1 and the chromatograms of xanthones in Figure S2.

Xanthone extracted from MP were calculated from the following equations [19,20]:

(2) $$\mathrm{Xanthone}\;\mathrm{concentration}\;\mathrm{in}\;\mathrm{MPE},\;\mathrm{mg}/g\;=\;\frac{mass\;of\;xanthone}{mass\;of\;MPE}$$

(3) $$\mathrm{Xanthone}\;\mathrm{extracted}\;\mathrm{from}\;\mathrm{MP},\;\mathrm{mg}/g\;=\;\frac{xanthone\;concentration\;\times \;mass\;of\;MPE}{mass\;of\;MP}$$

(4) $$\mathrm{Xanthone}\;\mathrm{selectivity}\;=\;\frac{mass\;of\;xanthones\;\times \;100}{mass\;of\;MPE\;-\;mass\;of\;xanthones}\%$$

(5) $$\mathrm{Co-extractant}\;\mathrm{effectiveness}\;=\;\frac{mass\;of\;xanthones\;\times \;100}{mass\;of\;co-extractant}\%$$

### 2.4. Viscosity Analysis

The viscosities of C10, C8, and their mixture (36% C10–64% C8) were measured with a rotational rheometer (MCR302, Anton Paar, Graz, Austria). Shear scanning was conducted in cylindrical mode over a shear rate range of (0.1 to 100) 1/s. The measurement was performed across a solvent temperature range of 25 °C to 100 °C, and the preheating time was 30 min [28].

### 2.5. Extraction Kinetics Modelling and Generalization

#### 2.5.1. Correlation with Pseudo-First-Order Kinetic Model

The extraction curves of yield over time were correlated using a pseudo-first-order kinetic model to describe the rate of solute transfer under scCO_2_ conditions with C8/C10 as a co-extractant. The equation is written as follows:

(6) $$Y\left(t\right)\;=\;Y_{max}\left(1\;-\;e^{-kt}\right)$$

where *Y*(*t*) is the extraction yield (%) at time *t* (min), *Y_max_* is the maximum extraction yield (%), and *k* is the rate constant (min^−1^). Fitting was performed using Microsoft Excel Solver, where the sum of squared errors (SSE) between experimental and predicted values was minimized as the objective function.

#### 2.5.2. Development of Generalized Predictive Models

To enable predictive modeling across different processing conditions, the fitted kinetic parameters *Y_max_* and *k* further correlated with pressure (*P*) and temperature (*T*) using second-order polynomial regression, written as follows:

(7) $$Z\;=\;\beta_{0}\;+\;\beta_{1}T\;+\;\beta_{2}P\;+\;\beta_{3}T^{2}\;+\;\beta_{4}P^{2}\;+\;\beta_{5}TP$$

where *Z* represents either *Y_max_* or *k*, and *β*_0_ to *β*_5_ are the regression coefficients. The second-order model was developed to account for both the individual and combined effects of pressure and temperature on the extraction behavior. Regression analysis was performed using the LINEST function in Microsoft Excel, and model performance was evaluated based on R^2^ and adjusted R^2^.

### 2.6. Statistical Analysis

The results were analyzed using analysis of variance (ANOVA) at a 5% uncertainty level using jamovi software (version 2.3.28, Sydney, Australia). The post hoc test was applied to determine the significant difference between the extraction methods.

## 3. Results and Discussion

### 3.1. Effect of C8/C10 Co-Extractant Ratio to Mangosteen Pericarp on Xanthone Extraction

Table 1 shows the effect of the co-extractant ratio on yield and xanthone extraction at 250 bar and 60 °C. Under these mild extraction conditions, the limited solubility of xanthones in scCO_2_ was expected to make the effect of co-extractants on enhancing recovery more apparent. Eight major xanthone monomers were identified, including garcinone D, garcinone C, β-mangostin, 8-deoxygartanin, gartanin, 1-isomangostin, α-mangostin, and γ-mangostin. Among these, α-mangostin was the predominant compound, consistent with previous reports in the literature [27,29]. Extraction yield and total xanthone concentration increased as the co-extractant–MP ratio increased. This trend was observed because a higher co-extractant ratio can improve the solubility of xanthones, leading to greater extraction efficiency. However, further analysis of the extracts revealed that not all targeted xanthones increased as the co-extractant ratio increased. For example, isomanostin, garcinone, and γ-mangostin did not follow this trend. In contrast, the concentration of α-mangostin, the dominant xanthone in MP, increased with a higher co-extractant ratio.

In Table 2 and Table 3, the 20% co-extractant ratio was used as the reference point, as it represents the lowest loading tested and allows clear comparison of enhancements at higher ratios. Pure scCO_2_ was not used as a baseline due to the extremely low xanthone yield and the absence of detectable isomangostin (Table 1), which would result in undefined or disproportionate ratios. As shown in Table 2, increasing the co-extractant beyond 20% generally led to improved effectiveness. However, the effectiveness of the co-extractant did not exhibit a linear relationship with xanthone extraction as the co-extractant ratio increased. Therefore, the suitable co-extractant ratio depends on the specific xanthone being targeted. Even though the co-extractant–MP ratio was directly proportional to the yield and total xanthones content (Table 1), especially at 40% co-extractant, the selectivity for specific xanthones decreased by up to 27% (Table 3, garcinone D). This effect might be due to the dilution effect of the co-extractant amount rather than its role in assisting xanthone extraction from the MP matrix. As for isomangostin, garcinone, and γ-mangostin, a 30% co-extractant ratio was found to be the most suitable.

**Figure 2:**
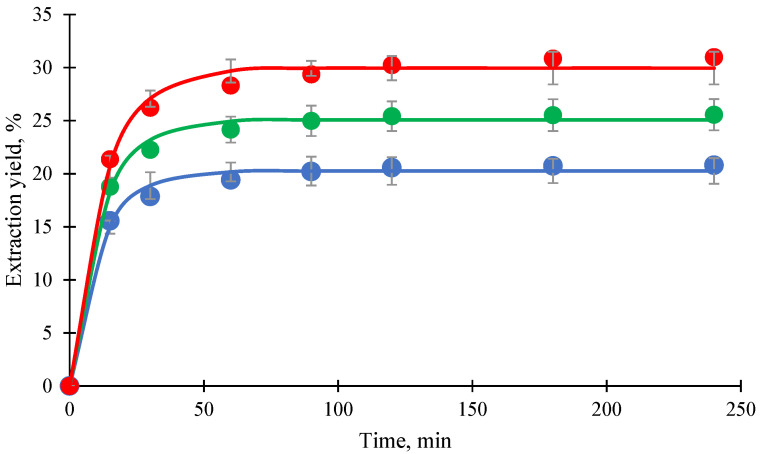
Extraction profiles of mangosteen pericarp extract using different C8/C10:mangoseen pericarp ratios at 250 bar, 60 °C; tricaprylin (C8):tricaprin (C10) = 0.64:0.36 in mass fraction; markers are experimental data: ●: C10/C8 to MP (0.2:0.8) (*w*/*w*), ●: C10/C8 to MP (0.3:0.7) (*w*/*w*), ●: C10/C8 to MP (0.4:0.6) (*w*/*w*); lines: pseudo-first-order kinetic model.

Figure 2 shows the extraction profiles of mangosteen pericarp extract using different C8/C10–MP ratios at 250 bar and 60 °C. All profiles exhibited a single characteristic trend, beginning with a rapid extraction phase within the first 15 min, followed by a gradual decline in extraction rate as equilibrium approached. During the initial phase, there were no significant differences in extraction yields that were observed among the various C8/C10 ratios. However, after 15 min, higher co-extractant ratios, particularly 40%, resulted in notably greater extraction yields. This trend suggests that increasing the C8/C10 ratio enhances solute diffusion and mass transfer efficiency, likely by improving the solvent’s ability to solubilize xanthones from the pericarp matrix.

The correlation of pseudo-first-order kinetic model (solid lines) with the experimental data (markers) across all tested co-extractant ratios is shown in Figure 2. The kinetic parameters derived from the model are summarized in Table 4. The maximum extraction yield (*Y_max_*) increased from 20.26% at 20% C8/C10 to 29.96% at 40%, confirming the enhanced extraction efficiency at higher co-extractant levels. Interestingly, the rate constant (*k*) decreased from 0.090 min^−1^ to 0.078 min^−1^ as the co-extractant ratio increased, suggesting increased mass transfer resistance at higher oil concentrations. According to Ilieva et al. [30], the solubility of scCO_2_ in vegetable oils can reach up to approximately 30%, significantly reducing the viscosity of the oil phase due to the plasticizing effect of dissolved CO_2_. However, once the oil becomes saturated with scCO_2_, further addition of oil (e.g., 40% C8/C10) does not contribute to additional viscosity reduction. Instead, the excess undissolved oil results in a more viscous co-extractant phase. This localized increase in viscosity may impede solute diffusion and elevate interfacial mass transfer resistance, thereby explaining the observed reduction in the kinetic rate constant at higher co-extractant ratios in this study. In a more viscous environment, the interaction between CO_2_ and the target solute may be reduced, while its capacity to solubilize or transport non-target components may be enhanced [31]. Consequently, scCO_2_ becomes less effective in selectively extracting the desired compounds compared to conditions with lower co-extractant concentrations. As observed in Table 3, selectivity for most xanthones declined at 40% co-extractant, supporting this interpretation.

Although the 40% C8/C10 ratio resulted in the highest *Y_max_*, it exhibited a slightly lower *k* compared to lower ratios. This outcome suggests a trade-off between extraction efficiency and mass transfer efficiency. Therefore, the 40% C8/C10–MP ratio, which produced the highest total xanthone concentration, was selected for subsequent experiments comparing different co-extractants.

### 3.2. Effect of Type of Co-Extractant on Xanthone Extraction

Table 5 compares different co-extractants for xanthone extraction from MP. The highest extraction yield was obtained with the C8/C10, followed by C8, C10, VCO, and ethanol. Similarly, the highest total xanthones content was also observed with C8/C10, followed by VCO, C8, ethanol, and C10. As reported by Liu et al. [23], the mixture of C8 and C10 exhibit a synergistic effect in solubilizing xanthones. This synergistic effect was also evident in the present study, resulting in higher extraction yield and total xanthone content compared to C8 or C10 alone. While VCO is also a triglyceride mixture with a high proportion of C8 and C10 [32,33,34], the synergistic effect of the defined C8/C10 mixture was more pronounced, resulting in higher total xanthones content than C8, C10, or ethanol.

Compared to oil-based co-extractants, particularly the C8/C10, both the extraction yield and total xanthones content obtained with ethanol were considerably lower. A similar trend was reported by Yao et al. (2025) [35], who observed higher xanthone concentrations when a VCO-ethanol solvent mixture was used in conjunction with ultrasound-assisted extraction from MP. This consistency suggests that oil-based co-extractants may offer superior solubilizing power for xanthones compared to polar solvents like ethanol, regardless of the extraction technique employed. This can be attributed to the predominantly hydrophobic nature of the xanthone core, composed of a fused aromatic ring system, which limits its solubility in polar environments [36]. In contrast, the C8/C10 mixture, consisting of medium-chain triglycerides, provides a lipophilic environment that promtotes hydrophobic interactions with the xanthone backbone, thereby enhancing solubility and extraction efficiency.

**Figure 3:**
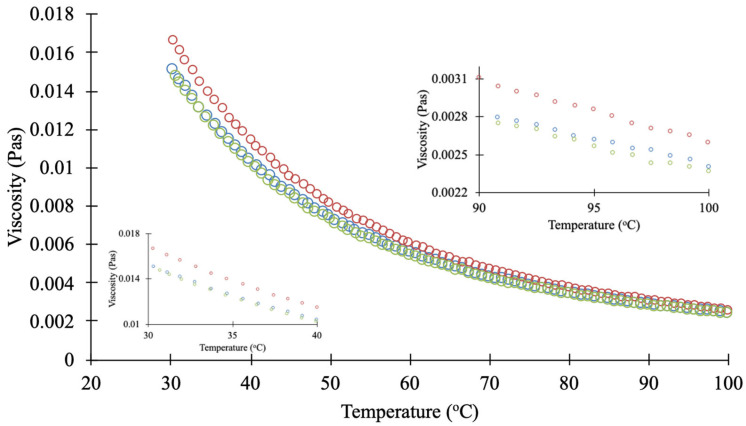
Dynamics viscosity of tricaprylin (C8, ◯), tricaprin (C10, ◯) and their mixture (0.64:0.36 in mass fraction, ◯).

Between C8 and C10, higher yield and total xanthone content were observed with C8. This trend was likely due to the lower dynamic viscosity of C8 compared to C10 (Figure 3), as C8 has a smaller molecular weight. Although both solvents share the same functional group, their differences in molecular size and viscosity may have influenced the extraction efficiency.

Generally, most of the targeted compounds were detected in the extracts obtained using all co-extractants. However, the extract obtained from C8/C10 contained the highest concentration of all targeted compounds, and isomangostin was detected only in the extract from C8/C10 (Table 5). In terms of effectiveness (Table 6) and selectivity (Table 7) for xanthones, oil-based co-extractants had distinct advantages for specific xanthones. For example, C8/C10 was suitable for isomangostin, γ-mangostin, and α-mangostin; C8 was suitable for garcinones; and VCO was suitable for 8-deoxygartanin, gartanin and β-mangostin. However, ethanol showed relatively lower effectiveness and selectivity than oil-based co-extractants, possibly due to its polarity, which may have promoted the extraction of other polar compounds from MP. These findings was in the agreement with the report from Walker [25]. Sungpud et al. [37] extracted polyphenols from MP using propylene glycol, VCO, and their mixture as solvents. It was found that the main components of the VCO extract were xanthones, including α-mangostin and γ-mangostin, whereas the propylene glycol extract also contained rutin, vanillic acid, epicatechin, and trans-ferulic acid in additional to xanthones. This demonstrates that the higher polarity of propylene glycol led to the extraction of more impurities. In summary, based on both extraction yield and total xanthone content, the C8/C10 was selected as the most effective co-extractant for subsequent stages of this study.

### 3.3. Effect of Different Supercritical Carbon Dioxide Conditions on Xanthone Extraction from Mangosteen Pericarp

**Figure 4:**
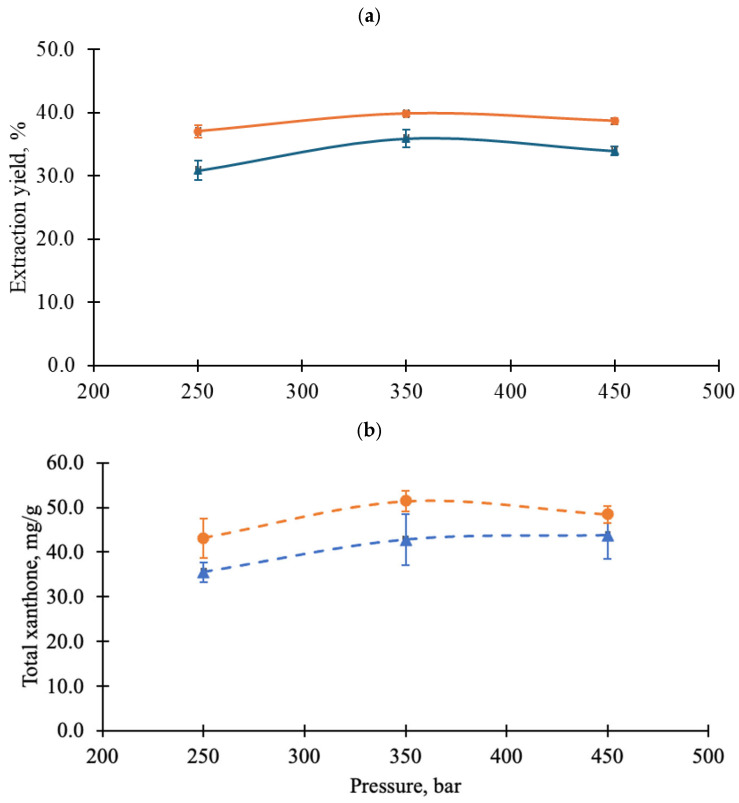
(**a**) Extraction yield and (**b**) total xanthones content in the mangosteen pericarp (MP) extract obtained various supercritical carbon dioxide conditions. ●: 70 °C; ▲: 60 °C; tricaprylin (C8)–tricaprin (C10) = 0.64:0.36 in mass fraction; co-extractant–MP (40%, *w*/*w*).

Figure 4 shows the extraction yield and total xanthones content in the extract obtained from various scCO_2_ conditions. The extraction yield (Figure 4a) and total xanthones content (Figure 4b) exhibited a concave trend, with a maximum observed at 350 bar for both 60 °C and 70 °C. The concave trend observed indicates that scCO_2_ acts as a selective solvent due to changes in its thermophysical properties under varying extraction conditions. At a given temperature, such as 60 °C, the density and viscosity of scCO_2_ increase with rising pressure (Table S6). As explained by Kok et al. [21] regarding the extraction mechanism of scCO_2_-VCO in xanthone extraction from MP, scCO_2_ and VCO penetrate the MP matrix to dissolve xanthones. As shown in Figure 4, the extraction yield and total xanthones content increased from 250 bar to 350 bar, the density of scCO_2_ increases from 786.6 kg/m^3^ to 862.9 kg/m^3^, which enhancing the penetration and solvation processes. With the increase in pressure, on the one hand, the solubility of xanthones in scCO_2_ increases, mainly because the density of scCO_2_ increases, which increases the number of collisions and binding probability between solute molecules and solvent molecules, so that solvent molecules take away more solutes and increase the solubility of solutes in scCO_2_ [38,39]. However, when the pressure increased from 350 bar to 450 bar, the viscosity of scCO_2_ increased from 84.6 mPa·s to 96.2 mPa·s. This is because with the increase in pressure, the distance between molecules decreases, and the cohesion between molecules increases, resulting in the increase in viscosity [40]. The viscosity of scCO_2_ may have played a more dominant role than density in xanthone extraction from MP. At high viscosity, scCO_2_ cannot diffuse efficiently, which may hinder mass transfer and reduce extraction efficiency.

At a given pressure, the extraction yield and total xanthones content obtained at 70 °C were higher than those at 60 °C (Figure 4). The enhancement at 70 °C can be attributed to increased molecular movement at higher temperatures [41]. As shown in Table S6, the density of scCO_2_ decreases when the temperature increases from 60 °C to 70 °C, which may reduce its solvating power for extracting xanthones from MP. However, this effect is counter-balanced by a simultaneous reduction in viscosity, which enhances mass transfer [42,43]. Therefore, in this study, viscosity played a more significant role in the extraction process. Temperature is also expected to affect the properties of C8/C10 co-extractant. As shown in Figure 3, the viscosity of C8/C10 decreased exponentially as the temperature increased from 30 °C to 100 °C. This reduction in viscosity is expected to enhance diffusivity into the MP matrix, facilitating xanthone extraction. Additionally, based on the Clausius-Clapeyron relationship [44], elevated temperatures increase the vapor pressure of xanthones, potentially enhancing their volatility and thermodynamic partitioning into the scCO_2_ phase [45], which may further contribute to the higher extraction observed at 70 °C. Statistical analysis confirmed that both extraction yield and total xanthone content at 70 °C were significantly higher than those at 60 °C (*p* < 0.05). Given the superior performance at 70 °C, pressure effects were further evaluated at this temperature. At 70 °C, the extraction yield at 350 bar was significantly greater than at 450 bar, and the total xanthone content was significantly higher at 350 bar compared to 250 bar (*p* < 0.05).

Table 8 and Table 9 summarize the effects of scCO_2_ pressure and temperature on the effectiveness and selectivity of individual xanthones, using 250 bar and 60 °C as the reference condition as the lowest pressure and temperature tested in this study. No consistent trends were observed across all xanthones; while some compounds exhibited increasing or decreasing responses, others followed concave or convex patterns. These observations were based on three data points per condition, which provided preliminary insight but may require additional measurements for robust trend analysis. Nevertheless, the data offer valuable guidance for identifying optimal extraction conditions for specific xanthones. For instance, α-mangostin (the major xanthone in MP) exhibited the highest extraction effectiveness at 350 bar and 70 °C, while maintaining selectivity comparable to that at 60 °C. This combination of enhanced recovery and stable selectivity supports 350 bar and 70 °C as the most suitable condition for its targeted extraction.

**Figure 5:**
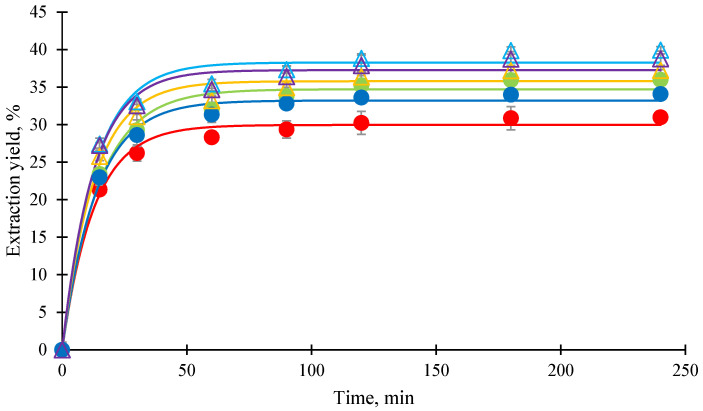
Extraction profiles of mangosteen pericarp extract at various supercritical carbon dioxide condition extract using tricaprylin (C8)–tricaprin (C10) = 0.64:0.36 in mass fraction as co-extractant; C8/C10–MP = 40% (*w*/*w*); markers are experimental data: ●: 250 bar, 60 °C, ●: 350 bar, 60 °C, ●: 450 bar, 60 °C, △: 250 bar, 70 °C, △: 350 bar, 70 °C, △: 450 bar, 70 °C; lines: pseudo-first-order kinetic model.

Figure 5 shows the extraction profiles of MPE under different scCO_2_ conditions. All curves exhibited similar trends, beginning with a rapid extraction phase lasting up to 15 min, likely extracting the more easily extractable solutes. This was followed by a declining extraction rate until 90 min, corresponding to the extraction of solutes from the deeper matrix. After 90 min, the extraction plateaued, indicating the transition into the diffusion phase, where C8/C10 may also have been depleted. However, differences in extraction yield were observed. The extraction profiles at 70 °C were higher than those at 60 °C. Similarly, extraction at moderate pressure (350 bar) resulted in the highest yield compared to 250 bar and 450 bar. As discussed in the previous section, high temperature (70 °C) and 350 bar enhanced diffusion into the MP matrix at a faster rate, leading to more effective xanthone extraction from the beginning to the end of the process, when the highest xanthone yield (39.93 ± 0.37%) was obtained. This improvement was strongly related to the reduced viscosity and surface tension of the solvents (scCO_2_ and C8/C10) under scCO_2_ conditions.

Based on an estimation from Figure 5, the extraction process of xanthones from MP can be considered complete around 120 min, as nearly 90% of the total yield was obtained by this time.

### 3.4. Generalized Predictive Model of Extraction Yield and Kinetics

**Figure 6:**
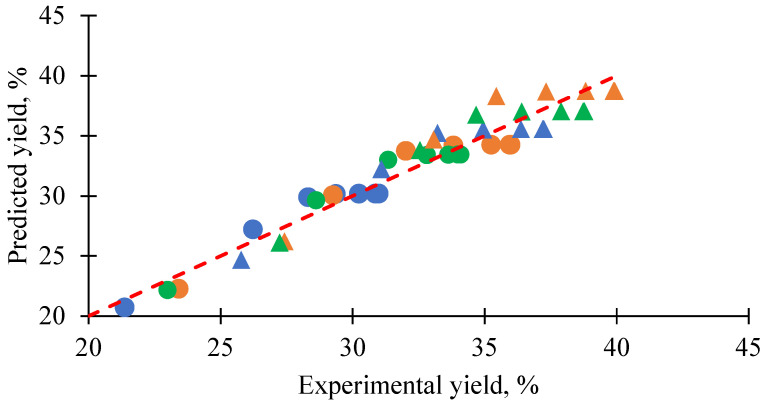
Experimental vs. predicted xanthone extraction yield from mangosteen pericarp using supercritical CO_2_ extraction with tricaprylin (C8) and tricaprin (C10) as a co-extractant (C8:C10 mass ratio = 0.64:0.36; C8/C10–MP = 40% *w*/*w*); predictions were made using second-order polynomial models for *Y_max_* (R^2^ = 0.981, adjusted R^2^ = 0.9304) and *k* = (R^2^= 0.9200, adjusted R^2^ = 0.5998); uncertainty = 2.3%; dashed red line = ideal 1:1 fit; markers: circles = 60 °C, triangles = 70 °C; blue = 250 bar, orange = 350 bar, green = 450 bar; extraction time = 240 min.

Eqn 8 and eqn 9 represented the generalized models develop based on experimental data obtained under varying pressure and temperature conditions studied in this work. These empirical models described the maximum yield (*Y_max_*) and rate constant (*k*) of the pseudo-first-order kinetic model. The model for *Y_max_* exhibited R^2^ = 0.9861 and adjusted R^2^ = 0.9304, while the model for *k* exhibited R^2^ = 0.9200 and adjusted R^2^ = 0.5998. The models were used to estimate the extraction yield, as shown in Figure 6. The predicted yield values deviated from the experimental results by an average uncertainty of ±2.3%, indicating good predictive accuracy. Despite the strong agreement observed in yield prediction, the model has certain limitations. The relatively small number of experimental data points (*n* = 6), compared to the number of fitted parameters, raised the potential for overfitting, particularly in the case of *k*. Therefore, the model was best suited for interpolation within the studied range rather than extrapolation beyond it.

(8) $$Y_{max,pre}\;=\;-22.38\;+\;0.2406P\;+\;5.84\;\times \;10^{-3}T^{2}\;-\;2.00\;\times \;10^{-3}P^{2}\;-\;9.00\;\times \;10^{-4}TP$$

(9) $$k_{pre}\;=\;0.158\;-\;1.00\;\times \;10^{-4}P\;-\;6.00\;\times \;10^{6}T^{2}\;+\;4.90\;\times \;10^{-7}P^{2}\;+\;3.58\;\times \;10^{-6}TP$$

### 3.5. Industry Relevance and Potential Expansion Strategy

The findings from this study provide a foundation for scaling up scCO_2_ extraction using C8/C10 as a sustainable alternative for xanthone recovery. C8/C10 is a food-grade co-extractant that does not require solvent separation and offers improved selectivity for specific xanthones while minimizing the extraction of unwanted impurities. Additionally, it aligns with clean-label and natural ingredient trends in the food and pharmaceutical industries [46], making it a promising method for producing high-purity xanthones with minimal processing contaminants. The xanthone concentration in the extract was approximately 5%, which may appear low; however, Pootakham et al. (2009) [47] reported that a formulation containing only 1% crude mangosteen extract remained stable and functional for 180 days. This suggests that the 5% xanthone-rich extract obtained in this study may be sufficiently concentrated for commercial applications.

However, we acknowledge that using 40% co-extractant may pose handling challenges at the industrial scale due to increased material volume and separation demands. Nonetheless, the low viscosity of C8/C10 remains advantageous in scCO_2_ extraction, particularly at elevated temperatures where it ensures good miscibility and fluidity. This enhances mass transfer, reduces flow resistance, and supports stable circulation through the extraction matrix. While higher co-extractant loading improves xanthone recovery, it also increases process complexity, a trade-off that must be considered in scale-up. A comprehensive techno-economic analysis, including CAPEX and OPEX considerations, is therefore recommended for future industrial implementation.

The generalized predictive models for extraction yield and kinetic rate constant further support scalability by enabling process optimization and control across a defined range of operating conditions, thereby facilitating informed decision-making during industrial implementation.

## 4. Conclusions

This study evaluated the use of C8 and C10 as co-extractants in scCO_2_ extraction for the recovery of xanthones from MP. The highest extraction yield and total xanthone content were achieved with a 40% C8/C10–MP ratio; however, selectivity decreased by approximately 15% compared to the 20% ratio, indicating a trade-off between extraction capacity and compound selectivity.

Among the tested co-extractants, the C8/C10 mixture exhibited the highest overall extraction efficiency and selectivity, particularly for α-mangostin. The synergistic effect of C8 and C10 in enhancing xanthone recovery was comparable to that observed in VCO, which naturally contains both components. Compared to ethanol, both C8/C10 and VCO demonstrated better performance in terms of selectivity and extraction efficiency.

Temperature and pressure were also found to significantly influence extraction behavior. Optimal conditions were identified at 70 °C and 350 bar, resulting in the highest total xanthone content. Furthermore, the selectivity for individual xanthones was shown to be tunable by adjusting the co-extractant type and operating parameters of the scCO_2_ system.

Additionally, pseudo-first-order kinetic modeling provided insights into mass transfer behavior, and the kinetic parameters were successfully generalized as functions of pressure and temperature using second-order polynomial equations. These models offer predictive capability for process design and optimization.

Overall, the findings underscore the potential of C8/C10 as a green and effective co-extractant for scCO_2_-based xanthone extraction. The optimized parameters established in this study offer a foundation for scale-up and application in the food, nutraceutical, and pharmaceutical industries.

## Figures and Tables

**Table 1:** Effect of ratio of co-extractant (C8/C10) to mangosteen pericarp on xanthone extraction from mangosteen pericarp.

C8/C10–MP (%, *w*/*w*)	Yield (%)	Total Xanthones (mg/g)	Isomangostin (mg/g)	Garcinone C (mg/g)	Garcinone D (mg/g)	8-Deoxygartanin (mg/g)	γ-Mangostin (mg/g)	Gartanin (mg/g)	α-Mangostin (mg/g)	β-Mangostin (mg/g)
20	20.80 ± 0.99 ^c^	22.61 ± 3.28 ^b^	0.03 ± 0.01 ^c^	0.19 ± 0.03 ^c^	0.28 ± 0.05 ^b^	0.84 ± 0.18 ^bc^	1.67 ± 0.21 ^b^	1.59 ± 0.34 ^ab^	17.44 ± 2.36 ^b^	0.58 ± 0.11 ^b^
30	25.56 ± 1.47 ^b^	30.09 ± 3.23 ^a^	0.05 ± 0.01 ^a^	0.59 ± 0.06 ^a^	0.49 ± 0.05 ^a^	1.00 ± 0.14 ^b^	3.26 ± 0.33 ^a^	1.87 ± 0.27 ^a^	22.14 ± 2.30 ^b^	0.69 ± 0.08 ^b^
40	30.90 ± ±1.52 ^a^	35.51 ± 2.21 ^a^	0.04 ± 0.00 ^b^	0.35 ± 0.02 ^b^	0.40 ± 0.02 ^a^	1.47 ± 0.10 ^a^	2.83 ± 0.19 ^a^	2.40 ± 0.15 ^a^	27.04 ± 1.67 ^a^	0.98 ± 0.06 ^a^
0	2.60 ± 0.04 ^d^	1.65 ± 0.16 ^c^	n.d.	0.01 ± 0.00 ^d^	0.01 ± 0.00 ^c^	0.15 ± 0.02 ^c^	0.06 ± 0.01 ^c^	0.30 ± 0.03 ^b^	1.03 ± 0.12 ^c^	0.07 ± 0.02 ^c^

Note: Experiment condition: 250 bar, 60 °C; tricaprylin (C8):tricaprin (C10) = 0.64:0.36 in mass fraction; total xanthones = summation of eight identified xanthones. The raw data are presented in Table S3. Within each group, samples labeled with different lowercase letters indicate significant differences at the *p* < 0.05 level.

**Table 2:** Effectiveness of co-extractant ratio on xanthone extraction from mangosteen pericarp.

	Co-Extractant Effectiveness (%)							
C8/C10–MP (%, *w*/*w*)	Isomangostin	Garcinone C	Garcinone D	8- Deoxygartanin	γ-Mangostin	Gartanin	α-Mangostin	β-Mangostin
20	100%	100%	100%	100%	100%	100%	100%	100%
30	192%	304%	174%	119%	196%	118%	127%	119%
40	146%	181%	145%	176%	169%	151%	155%	169%

Note: Comparisons were made based on 20% co-extractant usage; total xanthones = summation of eight identified xanthones. The raw data are presented in Table S4. (To facilitate a more precise comparative analysis of the results, the comparisons were standardized to 100%).

**Table 3:** Selectivity of co-extractant ratio on xanthone extraction from mangosteen pericarp.

	Selectivity (%)							
C8/C10–MP (%, *w*/*w*)	Isomangostin	Garcinone C	Garcinone D	8- Deoxygartanin	γ-Mangostin	Gartanin	α-Mangostin	β-Mangostin
20	100%	100%	100%	100%	100%	100%	100%	100%
30	136%	217%	124%	85%	140%	84%	90%	85%
40	74%	91%	73%	89%	85%	76%	77%	85%

Note: Comparisons were made based on 20% co-extractant usage; total xanthones = summation of eight identified xanthones. The raw data are presented in Table S5. (To facilitate a more precise comparative analysis of the results, the comparisons were standardized to 100%).

**Table 4:** Fitting parameters of pseudo-first-order kinetic model on effect of C8/C10 ratio to mangosteen pericarp (MP).

	C8/C10–MP		
	20%	30%	40%
*Y_max_* (%)	20.262	25.080	29.959
*k* (min^−1^)	0.090	0.086	0.078
SSE	2.590	2.367	5.358

Note: C8/C10–tricaprylin (C8): tricaprin (C10) = 0.64:0.36 in mass fraction; pseudo-first-order kinetic model, *Y_max_*: maximum extraction yield; *k*: rate constant; SSE: sum of square error.

**Table 5:** Effect type of co-extractant on xanthones extraction from mangosteen pericarp.

Co-Extractant	Yield (%)	Total Xanthones (mg/g)	Isomangostin (mg/g)	Garcinone C (mg/g)	Garcinone D (mg/g)	8- Deoxygartanin (mg/g)	γ-Mangostin (mg/g) *	Gartanin (mg/g)	α-Mangostin (mg/g)	β-Mangostin (mg/g)
C8/10	30.90 ± 1.52 ^a^	35.51 ± 2.21 ^a^	0.04 ± 0.00	0.35 ± 0.02 ^b^	0.40 ± 0.02 ^c^	1.47 ± 0.10 ^b^	2.83 ± 0.19 ^a^	2.40 ± 0.15 ^b^	27.04 ± 1.67 ^a^	0.98 ± 0.06 ^a^
C8	29.17 ± 0.98 ^ab^	27.52 ± 1.03 ^b^	n.d.	0.49 ± 0.01 ^a^	0.63 ± 0.01 ^a^	1.04 ± 0.05 ^c^	2.64 ± 0.07 ^a^	2.27 ± 0.12 ^bc^	19.84 ± 0.75 ^b^	0.62 ± 0.03 ^b^
C10	28.36 ± 1.24 ^ab^	17.31 ± 0.63 ^d^	n.d.	0.28 ± 0.01 ^c^	0.48 ± 0.01 ^b^	0.43 ± 0.03 ^d^	1.71 ± 0.05 ^b^	1.00 ± 0.06 ^d^	13.18 ± 0.46 ^c^	0.24 ± 0.01 ^d^
VCO	26.92 ± 1.00 ^b^	30.18 ± 1.34 ^b^	n.d.	0.09 ± 0.01 ^e^	0.31 ± 0.02 ^d^	2.53 ± 0.11 ^a^	1.48 ± 0.07 ^b^	4.93 ± 0.23 ^a^	19.88 ± 0.87 ^b^	0.96 ± 0.05 ^a^
Ethanol	28.87 ± 1.44 ^ab^	23.74 ± 1.14 ^c^	n.d.	0.13 ± 0.02 ^d^	0.34 ± 0.02 ^d^	0.89 ± 0.05 ^c^	1.50 ± 0.09 ^b^	1.92 ± 0.10 ^c^	18.49 ± 0.89 ^b^	0.48 ± 0.02 ^c^

Note: Experiment condition: 250 bar, 60 °C; tricaprylin (C8)–tricaprin (C10) = 0.64:0.36 in mass fraction; co-extractant–MP (40%, *w*/*w*); VCO: virgin coconut oil; n.d.: not detected; total xanthones = summation of eight identified xanthones. The data in the table were analyzed using one-way ANOVA in jamovi (Ver. 2.3) software. Post hoc tests were performed using Tukey’s HSD test for data with homogeneous variances, or the Games–Howell test for data with heterogeneous variances (marked with an asterisk after the group name). Within each group, samples labeled with different lowercase letters (a–e) indicate significant differences at the *p* < 0.05 level.

**Table 6:** Effectiveness of different co-extractant on xanthone extraction from mangosteen pericarp.

Effectiveness (%)								
Co-Extractant	Isomangostin	Garcinone C	Garcinone D	8-Deoxygartanin	γ-Mangostin	Gartanin	α-Mangostin	β-Mangostin
C8/10	100%	100%	100%	100%	100%	100%	100%	100%
C8	0%	138%	156%	71%	94%	94%	73%	63%
C10	0%	79%	119%	29%	60%	42%	49%	25%
VCO	0%	26%	76%	171%	52%	205%	74%	99%
Ethanol	0%	38%	83%	60%	53%	80%	68%	49%

Note: Experiment condition: 250 bar, 60 °C; tricaprylin (C8)–tricaprin (C10) = 0.64:0.36 in mass fraction; co-extractant–MP (40%, *w*/*w*); C8/C10 was used as basis for the comparison. (To facilitate a more precise comparative analysis of the results, the comparisons were standardized to 100%). The data is calculated based on the original data in Table S4 according to the formula mentioned above(the experimental data was in triplicate).

**Table 7:** Selectivity of different co-extractant on xanthone extraction from mangosteen pericarp.

	Selectivity (%)							
C8/C10–MP (%, *w*/*w*)	Isomangostin	Garcinone C	Garcinone D	8- Deoxygartanin	γ-Mangostin	Gartanin	α-Mangostin	β-Mangostin
C8/10	100%	100%	100%	100%	100%	100%	100%	100%
C8	0%	147%	166%	75%	99%	100%	77%	67%
C10	0%	86%	130%	32%	66%	45%	52%	27%
VCO	0%	29%	88%	198%	60%	237%	84%	114%
Ethanol	0%	41%	90%	64%	57%	86%	72%	52%

Note: Experiment condition: 250 bar, 60 °C; tricaprylin (C8):tricaprin (C10) = 0.64:0.36 in mass fraction; co-extractant–MP (40%, *w*/*w*); C8/C10 was used as basis for the comparison. (To facilitate a more precise comparative analysis of the results, the comparisons were standardized to 100%). The data is calculated based on the original data in Table S5 according to the formula mentioned above (the experimental data was in triplicate).

**Table 8:** Effectiveness of different supercritical carbon dioxide conditions on xanthone extraction from mangosteen pericarp.

		Effectiveness (%)							
Pressure (bar)	Temperature (°C)	Isomangostin	Garcinone C	Garcinone D	8-Deoxygartanin	γ-Mangostin	Gartanin	α-Mangostin	β-Mangostin
250	60	100%	100%	100%	100%	100%	100%	100%	100%
350	60	101%	94%	140%	75%	110%	97%	128%	74%
450	60	113%	111%	126%	86%	125%	106%	128%	95%
250	70	4%	48%	102%	174%	199%	96%	114%	136%
350	70	156%	147%	198%	91%	167%	114%	149%	101%
450	70	130%	113%	134%	93%	134%	106%	143%	105%

Note: tricaprylin (C8):tricaprin (C10) = 0.64:0.36 in mass fraction; co-extractant–MP (40%, *w*/*w*); 250 bar, 60 °C condition was used as basis for the comparison. (To facilitate a more precise comparative analysis of the results, the comparisons were standardized to 100%). The data is calculated based on the original data in Table S4 according to the formula mentioned above (the experimental data was in triplicate).

**Table 9:** Selectivity of different supercritical carbon dioxide conditions on xanthone extraction from mangosteen pericarp.

		Selectivity (%)							
Pressure (bar)	Temperature (°C)	Isomangostin	Garcinone C	Garcinone D	8-Deoxygartanin	γ-Mangostin	Gartanin	α-Mangostin	β-Mangostin
250	60	100%	100%	100%	100%	100%	100%	100%	100%
350	60	91%	85%	127%	68%	100%	88%	117%	68%
450	60	91%	90%	101%	70%	100%	85%	103%	76%
250	70	4%	42%	89%	152%	175%	84%	99%	119%
350	70	121%	114%	154%	71%	130%	88%	117%	78%
450	70	103%	89%	106%	73%	105%	83%	114%	83%

Note: tricaprylin (C8)–tricaprin (C10) = 0.64:0.36 in mass fraction; co-extractant–MP (40%, *w*/*w*); 250 bar, 60 °C condition was used as basis for the comparison. (To facilitate a more precise comparative analysis of the results, the comparisons were standardized to 100%). The data is calculated based on the original data in Table S5 according to the formula mentioned above (the experimental data was in triplicate).

## Data Availability

The original contributions presented in this study are included in the article. Further inquiries can be directed to the corresponding author.

## Supplementary Materials

The following supporting information can be downloaded at: https://www.mdpi.com/article/10.3390/foods14172983/s1, Table S1: Specifications of xanthone derivatives, tricaprylin, and tricaprin; Table S2: Fatty acid profiling of virgin coconut oil; Table S3: Content data of xanthones compounds extracted from MP by scCO_2_ with different co-extractants under different conditions; Table S4: Data of Co-extractant effectiveness with different co-extractants under different conditions; Table S5: Data of selectivity with different co-extractants under different conditions; Table S6: Thermophysical properties of supercritical carbon dioxide in this study; Figure S1: Calibration curve of the corresponding standard xanthone compounds; Figure S2: Chromatograms of xanthones.
