# Supplementary material for: Green Co-Extractant-Assisted Supercritical CO2 Extraction of Xanthones from Mangosteen Pericarp Using Tricaprylin and Tricaprin Mixtures
Source: Foods. 2025 Aug 26;14(17):2983. doi: 10.3390/foods14172983 (PMC12428359; doi:10.3390/foods14172983)
Supplement: Supplementary file 1 [file foods-14-02983-s001.zip › foods-3774611-supplementary.pdf]

Supplemental Material:

Green Co-Extractant-Assisted Supercritical CO_2 Extraction of Xanthones from Mangosteen Pericarp Using Tricaprylin and Tricaprin Mixtures

Hua Liu[1, 2], Johnson Stanslas[3], Jiaoyan Ren[4], Norhidayah binti Suleiman[2], Gun Hean Chong[2, 5*]

[1].Guangzhou College of Technology and Business, GuangZhou 510850, China

[2].Food Science and Technology, Universiti Putra Malaysia, Serdang 43400, Malaysia

[3]Medicine and Health Sciences, Universiti Putra Malaysia, Serdang 43400, Malaysia.

[4]Food Science and Engineering, South China University of Technology, GuangZhou 510000, China.

[5]Supercritical Fluid Centre (SFC), Faculty of Food Science and Technology, Universiti Putra Malaysia, 43400 Serdang, Selangor, Malaysia.

* Corresponding author, Email: gunhean@upm.edu.my

Table S1: Specifications of xanthone derivatives, tricaprylin, and tricaprin

Material	CAS	Mass fraction purity	Chemical molecular formula	Molar mass (g/mol)
garcinone D	107390-08-9	98.62	$C_{24}H_{28}O_7$	428.475
garcinone C	76996-27-5	99.34	$C_{23}H_{26}O_7$	414.448
β-mangostin	20931-37-7	98.86	$C_{25}H_{28}O_6$	424.490
8-deoxygartanin	33390-41-9	99.22	$C_{23}H_{24}O_5$	380.43
gartanin	33390-42-0	99.35	$C_{23}H_{24}O_6$	396.433
1-isomangostin	19275-44-6	99.41	$C_{24}H_{26}O_6$	410.46
α-mangostin	6147-11-1	99.09	$C_{24}H_{26}O_6$	410.46
γ-mangostin	31271-07-5	99.28	$C_{23}H_{24}O_6$	396.439
tricaprylin(C8)	538-23-8	99.00	$C_{27}H_{50}O_6$	498.1
tricaprin (C10)	621-71-6	99.00	$C_{33}H_{62}O_6$	554.8

Note: Purity values were provided by the supplier. Chromatograms of xanthones from this study were shown in Figure S1

Table S2: Fatty acid profiling of virgin coconut oil

Fatty acid composition	Molecular formula	Mass fraction (%)
Octanoic acid	$C_8H_{16}O_2$	8.13
Decanoic acid	$C_{10}H_{20}O_2$	5.52
Dodecanoic acid	$C_{12}H_{24}O$	46.97
Tetradecanoic acid	$C_{14}H_{28}O_2$	19.54
Hexadecanoic acid	$C_{16}H_{32}O_2$	7.91
Octadecanoic acid	$C_{18}H_{34}O_2$	4.65
Octadecanoic acid	$C_{18}H_{36}O_2$	3.12

0.1 g of the oil sample was weighed into a 10 mL test tube. The sample was mixed with 1 mL of 2 M potassium hydroxide (KOH) methanol solution and thoroughly vortexed to ensure complete dissolution. Following this, 5 mL of n-hexane was added, and the mixture was vortexed again and allowed to stand for phase separation. A 1 mL aliquot of the supernatant was then taken for gas chromatography (GC) analysis. The extracted fatty acids were methylated to form fatty acid methyl esters (FAMEs), which were analyzed using an HP-5MS elastic quartz capillary chromatography column (30 m × 0.25 mm × 0.25 μm). Helium was used as the carrier gas at a flow rate of 1.00 mL/min, with the injection port

maintained at 200°C. A non-split injection method was employed, and the column temperature was programmed to start at 40°C and ramp up to 300°C at 5°C/min. The composition of the fatty acids was determined using an in-house library of reference spectra.

Table S3: Content data of xanthones compounds extracted from MP by $scCO_2$ with different co-extractants under different conditions

Run	Extraction Conditions	Isomagostin (mg/g)	Garcinone C (mg/g)	Garcinone D (mg/g)	8-deoxygartanin (mg/g)	γ-mangostin (mg/g)	Gartanin (mg/g)	α-mangostin (mg/g)	β-mangostin (mg/g)	Xanthones (mg/g)
0	250bar,60℃, scCO2	n.d.	0.01±0.00	0.01±0.00	0.15±0.02	0.06±0.01	0.30±0.03	1.03±0.12	0.07±0.02	1.65±0.16
1	250bar,60℃, $scCO_2$-20%C8/C10	0.0260±0.0052	0.1937±0.0318	0.2785±0.0504	0.8364±0.1788	1.6684±0.2064	1.5873±0.3420	17.4380±2.3573	0.5792±0.1146	22.6074±3.2756
2	250bar,60℃, $scCO_2$-30%C8/C10	0.0498±0.0054	0.5881±0.0601	0.4851±0.0543	0.9956±0.1368	3.2626±0.3276	1.8729±0.2730	22.1413±2.2973	0.6900±0.0831	30.0853±3.2264
3	250bar,60℃, $scCO_2$-40%C8/C10	0.0380±0.0021	0.3513±0.0200	0.4035±0.0218	1.4749±0.0988	2.8266±0.1903	2.4042±0.1477	27.0356±1.6730	0.9769±0.0626	35.5111±2.2091
4	350bar,60℃, $scCO_2$-40%C8/C10	0.0382±0.0059	0.3301±0.0292	0.5653±0.0491	1.1108±0.1156	3.0987±0.1070	2.3336±0.2654	34.5841±1.7831	0.7270±0.0747	42.7879±5.6478
5	350bar,70℃, $scCO_2$-40%C8/C10	0.0593±0.0023	0.5168±0.0304	0.7995±0.0315	1.3418±0.0579	4.7112±0.1792	2.7332±0.1287	40.2906±1.8036	0.9863±0.0389	51.4387±2.2239
6	450bar,60℃, $scCO_2$-40%C8/C10	0.0429±0.0008	0.3897±0.0325	0.5068±0.0304	1.2748±0.0867	3.5195±0.0864	2.5483±0.3122	34.5529±3.4396	0.9249±0.1390	43.7602±5.3596
7	450bar,70℃, $scCO_2$-40%C8/C10	0.0496±0.0022	0.3977±0.0316	0.5409±0.0429	1.3755±0.0514	3.7738±0.2953	2.5398±0.1437	38.7320±1.5227	1.0249±0.0211	48.4342±1.9311
8	250bar,70℃, $scCO_2$-40%C8/C104	0.0009±0.0001	0.1680±0.0210	0.4095±0.0151	2.5651±0.1624	5.6234±0.4323	2.3089±0.1150	30.7224±1.7715	1.3269±0.0923	43.1251±4.3434
9	250bar,60℃, $scCO_2$-40%ethanol	0.0000±0.0000	0.1336±0.0201	0.3366±0.0196	0.8851±0.0474	1.5012±0.0925	1.9163±0.1021	18.4932±0.8947	0.4767±0.0250	23.7427±1.1386
10	250bar,60℃, $scCO_2$-40%C10	0.0000±0.0000	0.2762±0.0108	0.4809±0.0116	0.4307±0.0303	1.7065±0.0474	0.9987±0.0611	13.1807±0.4648	0.2403±0.0136	17.3140±0.6348
11	250bar,60℃, $scCO_2$-40%VCO	0.0000±0.0000	0.0897±0.0104	0.3073±0.0158	2.5256±0.1079	1.4835±0.0681	4.9260±0.2288	19.8821±0.8665	0.9640±0.0486	30.1783±1.3372
12	250bar,60℃, $scCO_2$-40%C8	0.0000±0.0000	0.4862±0.0148	0.6297±0.0148	1.0421±0.0497	2.6441±0.0684	2.2677±0.1188	19.8363±0.7518	0.6184±0.0262	27.5246±1.0299

Table S4: Data of Co-extractant effectiveness with different co-extractants under different conditions

Run	Extraction Conditions	Isomagostin (mg/g)	Garcinone C (mg/g)	Garcinone D (mg/g)	8-deoxygartanin (mg/g)	γ-mangostin (mg/g)	Gatanin (mg/g)	α-mangostin (mg/g)	β-mangostin (mg/g)
1	250bar,60℃, $scCO_2$-20%C8/C10	0.0039±0.0008	0.0290±0.0048	0.0418±0.0076	0.1255±0.0268	0.2503±0.0310	0.2381±0.0513	2.6157±0.3536	0.0039±0.0008
2	250bar,60℃, $scCO_2$-30%C8/C10	0.0075±0.0008	0.0882±0.0090	0.0728±0.0081	0.1493±0.0205	0.4894±0.0491	0.2809±0.0409	3.3212±0.3446	0.0075±0.0008
3	250bar,60℃, $scCO_2$-40%C8/C10	0.0057±0.0003	0.0527±0.0033	0.0605±0.0014	0.2212±0.0011	0.4240±0.0020	0.3606±0.0135	4.0553±0.1224	0.0057±0.0003
4	350bar,60℃, $scCO_2$-40%C8/C10	0.0057±0.0009	0.0495±0.0044	0.0848±0.0074	0.1666±0.0173	0.4648±0.0160	0.3500±0.0398	5.1876±0.2675	0.0057±0.0009
5	350bar,70℃, $scCO_2$-40%C8/C10	0.0089±0.0003	0.0775±0.0046	0.1199±0.0047	0.2013±0.0087	0.7067±0.0269	0.4100±0.0193	6.0436±0.2705	0.0089±0.0003
6	450bar,60℃, $scCO_2$-40%C8/C10	0.0065±0.0001	0.0585±0.0049	0.0760±0.0046	0.1912±0.0130	0.5279±0.0130	0.3822±0.0468	5.1830±0.5159	0.0065±0.0001
7	450bar,70℃, $scCO_2$-40%C8/C10	0.0074±0.0003	0.0596±0.0047	0.0811±0.0064	0.2063±0.0077	0.5661±0.0443	0.3810±0.0216	5.8098±0.2284	0.0074±0.0003
8	250bar,70℃, $scCO_2$-40%C8/C104	0.0002±0.0000	0.0252±0.0032	0.0614±0.0023	0.3848±0.0244	0.8435±0.0648	0.3463±0.0172	4.6084±0.2657	0.0002±0.0000
9	250bar,60℃, $scCO_2$-40%ethanol	0.0000±0.0000	0.0200±0.0030	0.0505±0.0029	0.1328±0.0071	0.2252±0.0139	0.2874±0.0153	2.7740±0.1342	0.0000±0.0000
10	250bar,60℃, $scCO_2$-40%C10	0.0000±0.0000	0.0414±0.0016	0.0721±0.0017	0.0646±0.0045	0.2560±0.0071	0.1498±0.0092	1.9771±0.0697	0.0000±0.0000
11	250bar,60℃, $scCO_2$-40%VCO	0.0000±0.0000	0.0135±0.0016	0.0461±0.0024	0.3788±0.0162	0.2225±0.0102	0.7389±0.0343	2.9823±0.1300	0.0000±0.0000
12	250bar,60℃, $scCO_2$-40%C8	0.0000±0.0000	0.0729±0.0022	0.0945±0.0022	0.1563±0.0075	0.3966±0.0103	0.3402±0.0178	2.9755±0.1128	0.0000±0.0000

Table S5: Data of selectivity with different co-extractants under different conditions

Run	Extraction Conditions	Isomagostin (mg/g)	Garcinone C (mg/g)	Garcinone D (mg/g)	8-deoxygartanin (mg/g)	γ-mangostin (mg/g)	Gatanin (mg/g)	α-mangostin (mg/g)	β-mangostin (mg/g)
0	250bar,60℃, $scCO_2$	0.0000±0.0000	0.0594±0.0055	0.0563±0.0038	0.8507±0.0412	0.3386±0.0073	1.7161±0.0838	6.1492±0.3020	0.3951±0.0349
1	250bar,60℃, $scCO_2$-20%C8/C10	0.0100±0.0025	0.0745±0.0153	0.1072±0.0243	0.3227±0.0840	0.6458±0.1075	0.6143±0.1611	7.1891±1.3877	0.2232±0.0546
2	250bar,60℃, $scCO_2$-30%C8/C10	0.0136±0.0012	0.1614±0.0123	0.1331±0.0110	0.2735±0.0331	0.9019±0.0655	0.5158±0.0676	6.4579±0.5334	0.1894±0.0190
3	250bar,60℃, $scCO_2$-40%C8/C10	0.0074±0.0003	0.0681±0.0053	0.0782±0.0043	0.2864±0.0142	0.5503±0.0274	0.4677±0.0285	5.5235±0.3301	0.1895±0.0244
4	350bar,60℃, $scCO_2$-40%C8/C10	0.0067±0.0012	0.0581±0.0061	0.0996±0.0096	0.1959±0.0260	0.5485±0.0369	0.4125±0.0589	6.4835±0.4196	0.1282±0.0161
5	350bar,70℃, $scCO_2$-40%C8/C10	0.0089±0.0004	0.0777±0.0054	0.1203±0.0061	0.2020±0.0107	0.7130±0.0353	0.4124±0.0236	6.4449±0.3709	0.1484±0.0071
6	450bar,60℃, $scCO_2$-40%C8/C10	0.0067±0.0001	0.0609±0.0050	0.0793±0.0047	0.1996±0.0103	0.5530±0.0069	0.3998±0.0466	5.7076±0.5628	0.1447±0.0209
7	450bar,70℃, $scCO_2$-40%C8/C10	0.0076±0.0004	0.0607±0.0043	0.0825±0.0058	0.2101±0.0070	0.5786±0.0404	0.3887±0.0194	6.2747±0.3023	0.1565±0.0032
8	250bar,70℃, $scCO_2$-40%C8/C104	0.0003±0.0000	0.0284±0.0044	0.0693±0.0030	0.4360±0.0284	0.9607±0.1037	0.3922±0.0199	5.4840±0.2653	0.2250±0.0223
9	250bar,60℃, $scCO_2$-40%ethanol	0.0000±0.0000	0.0278±0.0042	0.0700±0.0027	0.1843±0.0040	0.3130±0.0141	0.3999±0.0087	3.9975±0.0260	0.0992±0.0019
10	250bar,60℃, $scCO_2$-40%C10	0.0000±0.0000	0.0585±0.0035	0.1018±0.0043	0.0912±0.0040	0.3623±0.0142	0.2117±0.0084	2.8686±0.1062	0.0509±0.0018
11	250bar,60℃, $scCO_2$-40%VCO	0.0000±0.0000	0.0200±0.0017	0.0685±0.0013	0.5661±0.0119	0.3318±0.0050	1.1101±0.0269	4.6369±0.0667	0.2153±0.0051
12	250bar,60℃, $scCO_2$-40%C8	0.0000±0.0000	0.1001±0.0029	0.1297±0.0045	0.2148±0.0148	0.5468±0.0213	0.4686±0.0330	4.2532±0.2299	0.1274±0.0081

Table S6: Thermophysical properties of supercritical carbon dioxide in this study

Pressure (bar)	Temperature (°C)	Density (kg/ m^3)	Viscosity (mPa·s)
250	60	786.6	70.2
350	60	862.9	84.6
450	60	913.3	96.2
250	70	736.9	62.7
350	70	826.1	77.5
450	70	882.4	89.1

Source: NIST Chemistry WebBook

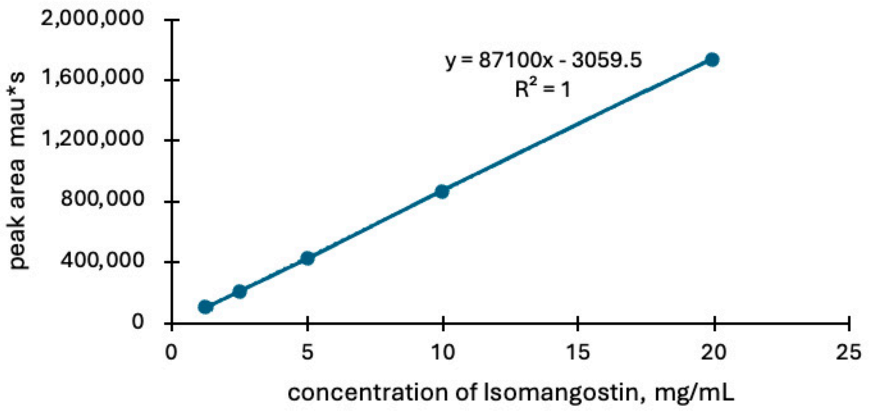
2,000,000
1,600,000
1,200,000
800,000
400,000
0
peak area mau*s
y = 87100x - 3059.5
R^2 = 1
0
5
10
15
20
25
concentration of Isomangostin, mg/mL

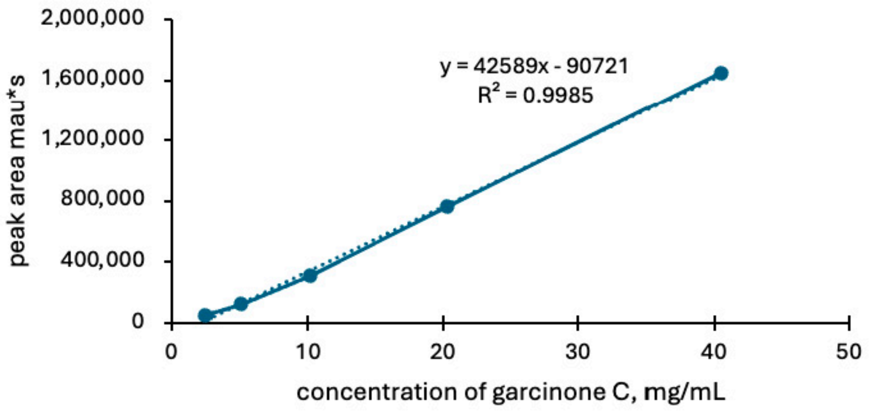
2,000,000
1,600,000
1,200,000
800,000
400,000
0
peak area mau*s
y = 42589x - 90721
R^2 = 0.9985
0
10
20
30
40
50
concentration of garcinone C, mg/mL

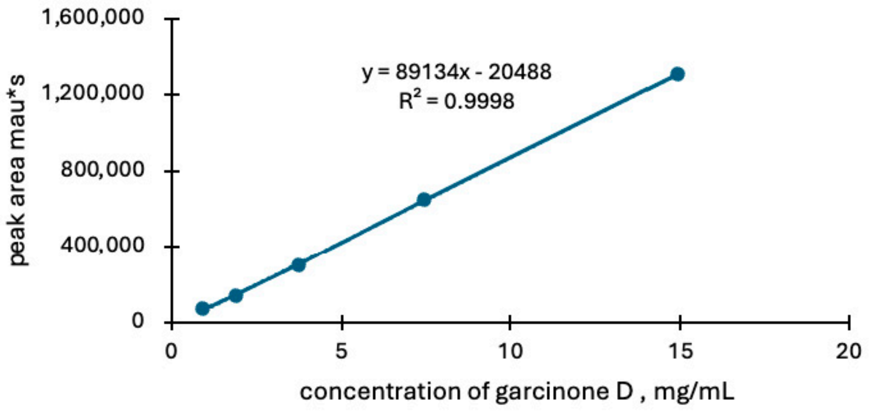
1,600,000
1,200,000
800,000
400,000
0
peak area mau*s
y = 89134x - 20488
R^2 = 0.9998
0
5
10
15
20
concentration of garcinone D , mg/mL

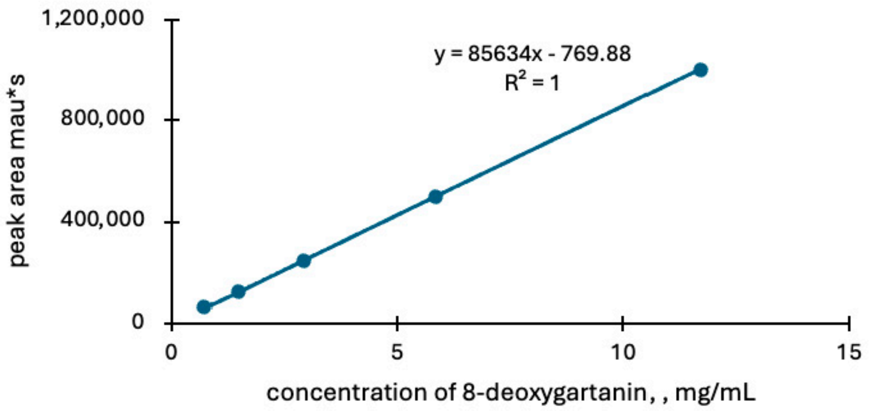
y = 85634x - 769.88
R² = 1
peak area mau*s
concentration of 8-deoxygartanin, , mg/mL

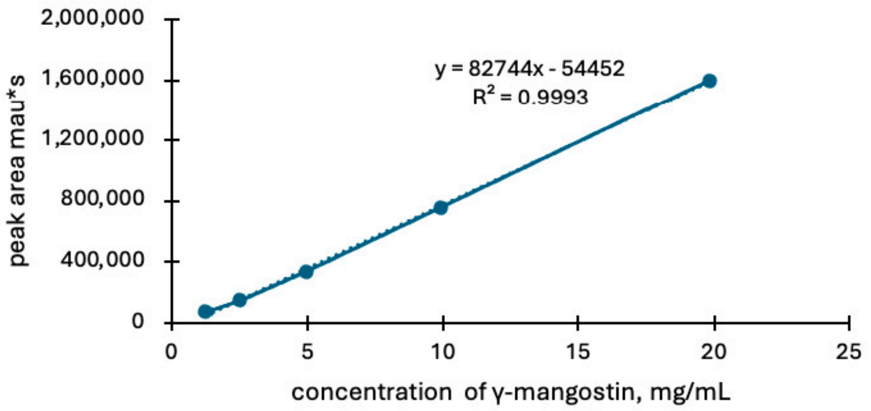
y = 82744x - 54452
R² = 0.9993
peak area mau*s
concentration of γ-mangostin, mg/mL

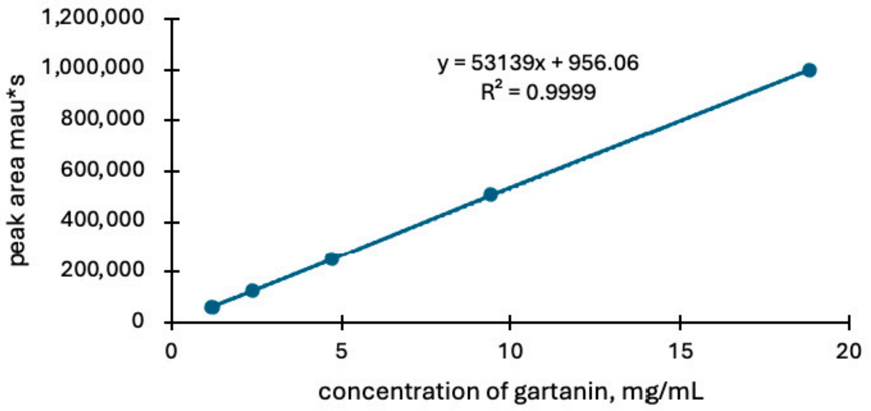
y = 53139x + 956.06
R² = 0.9999
peak area mau*s
concentration of gartanin, mg/mL

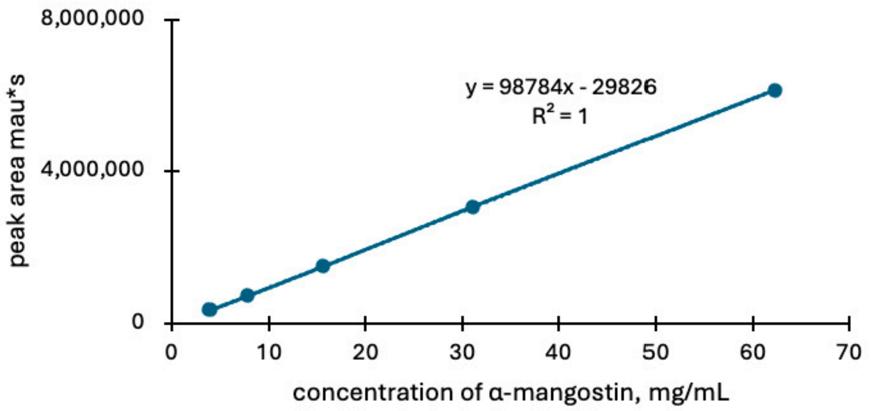

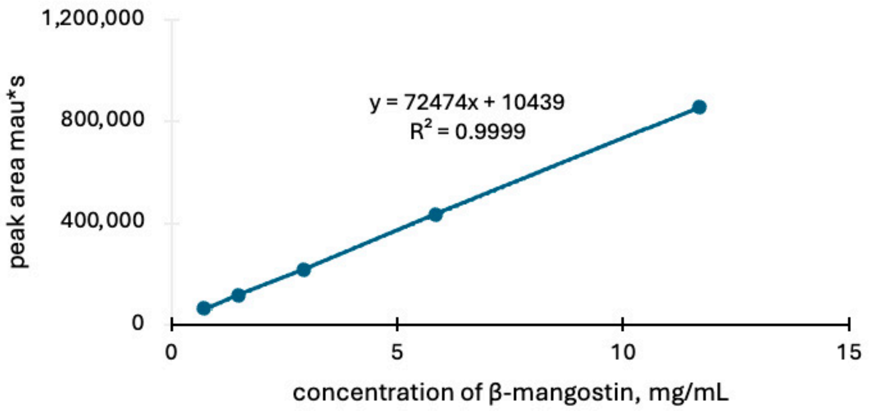

Figure S1: Calibration curve of the corresponding standard xanthone compounds

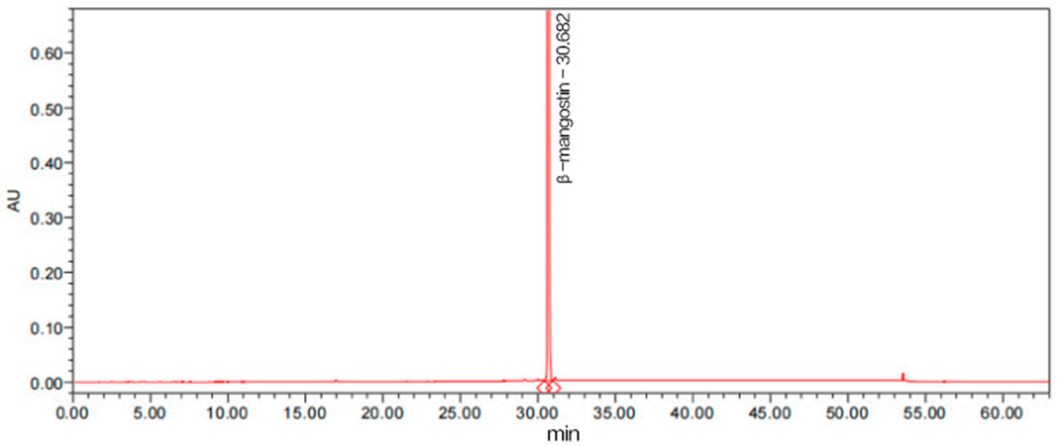
β −mangostin − 30.682
AU
0.60
0.50
0.40
0.30
0.20
0.10
0.00
0.00 5.00 10.00 15.00 20.00 25.00 30.00 35.00 40.00 45.00 50.00 55.00 60.00
min

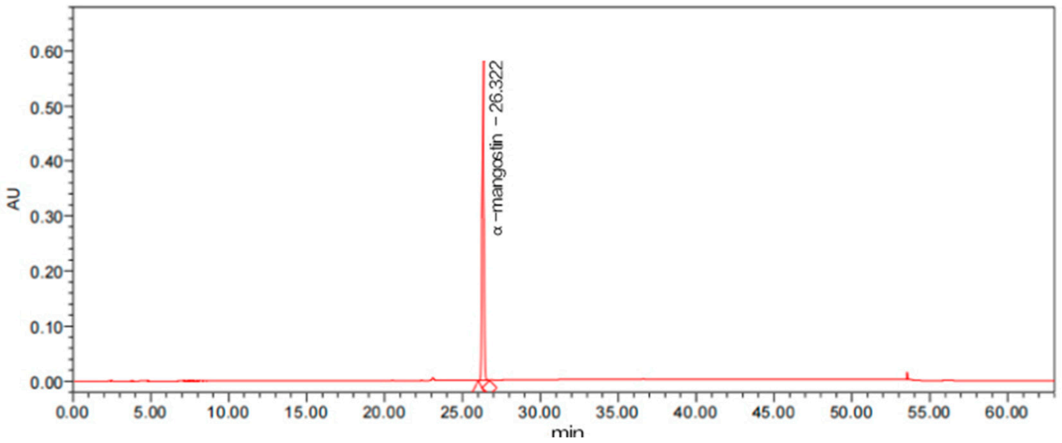
α −mangostin − 26.322
AU
0.60
0.50
0.40
0.30
0.20
0.10
0.00
0.00 5.00 10.00 15.00 20.00 25.00 30.00 35.00 40.00 45.00 50.00 55.00 60.00
min

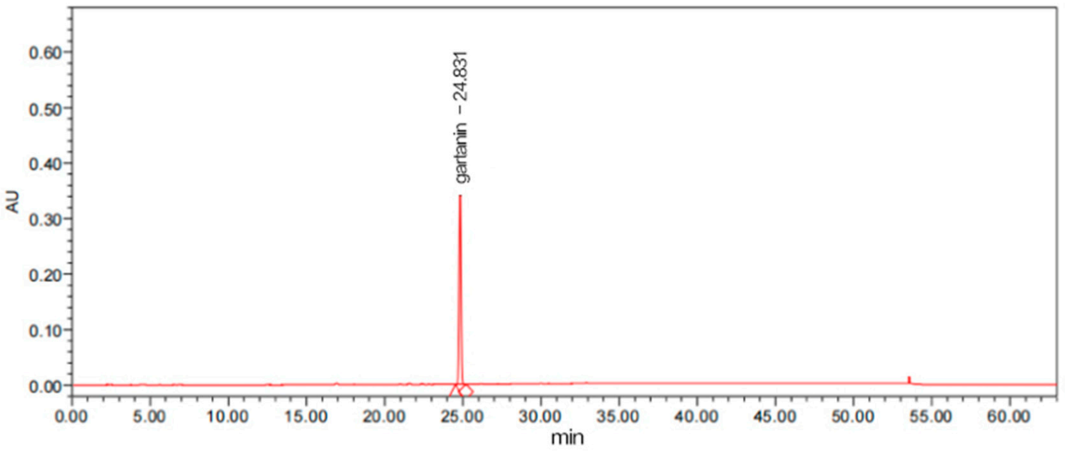
gartanin − 24.831
AU
0.60
0.50
0.40
0.30
0.20
0.10
0.00
0.00 5.00 10.00 15.00 20.00 25.00 30.00 35.00 40.00 45.00 50.00 55.00 60.00
min

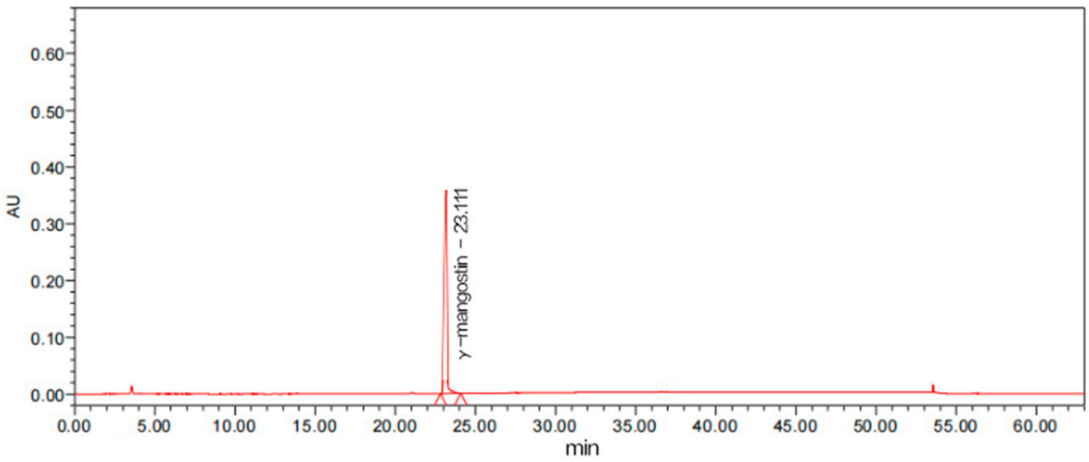

AU
0.60
0.50
0.40
0.30
0.20
0.10
0.00
γ-mangostin - 23.111
0.00 5.00 10.00 15.00 20.00 25.00 30.00 35.00 40.00 45.00 50.00 55.00 60.00
min

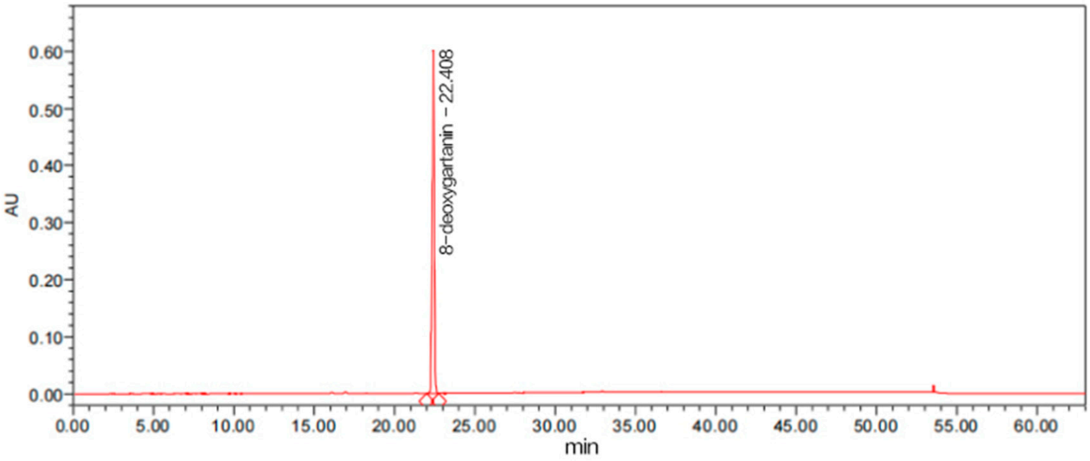

AU
0.60
0.50
0.40
0.30
0.20
0.10
0.00
8-deoxygartanin - 22.408
0.00 5.00 10.00 15.00 20.00 25.00 30.00 35.00 40.00 45.00 50.00 55.00 60.00
min

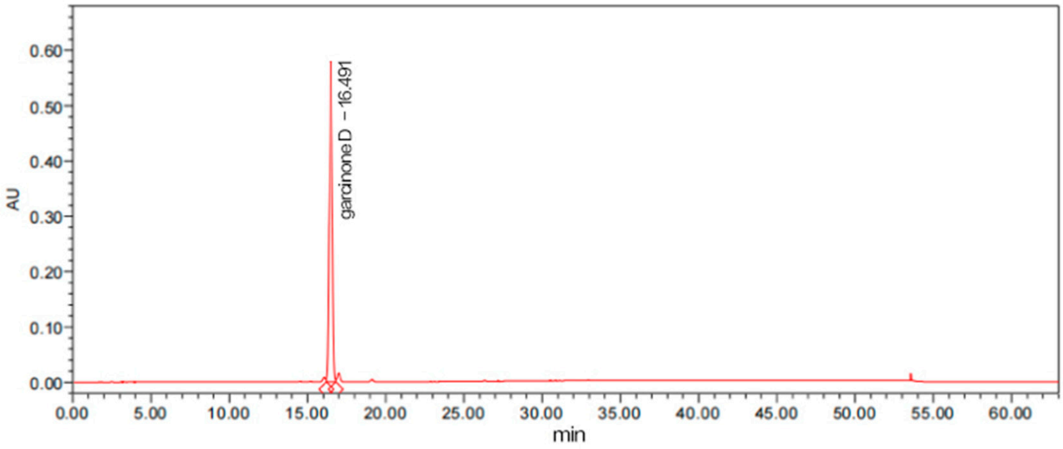

AU
0.60
0.50
0.40
0.30
0.20
0.10
0.00
garcinone D - 16.491
0.00 5.00 10.00 15.00 20.00 25.00 30.00 35.00 40.00 45.00 50.00 55.00 60.00
min

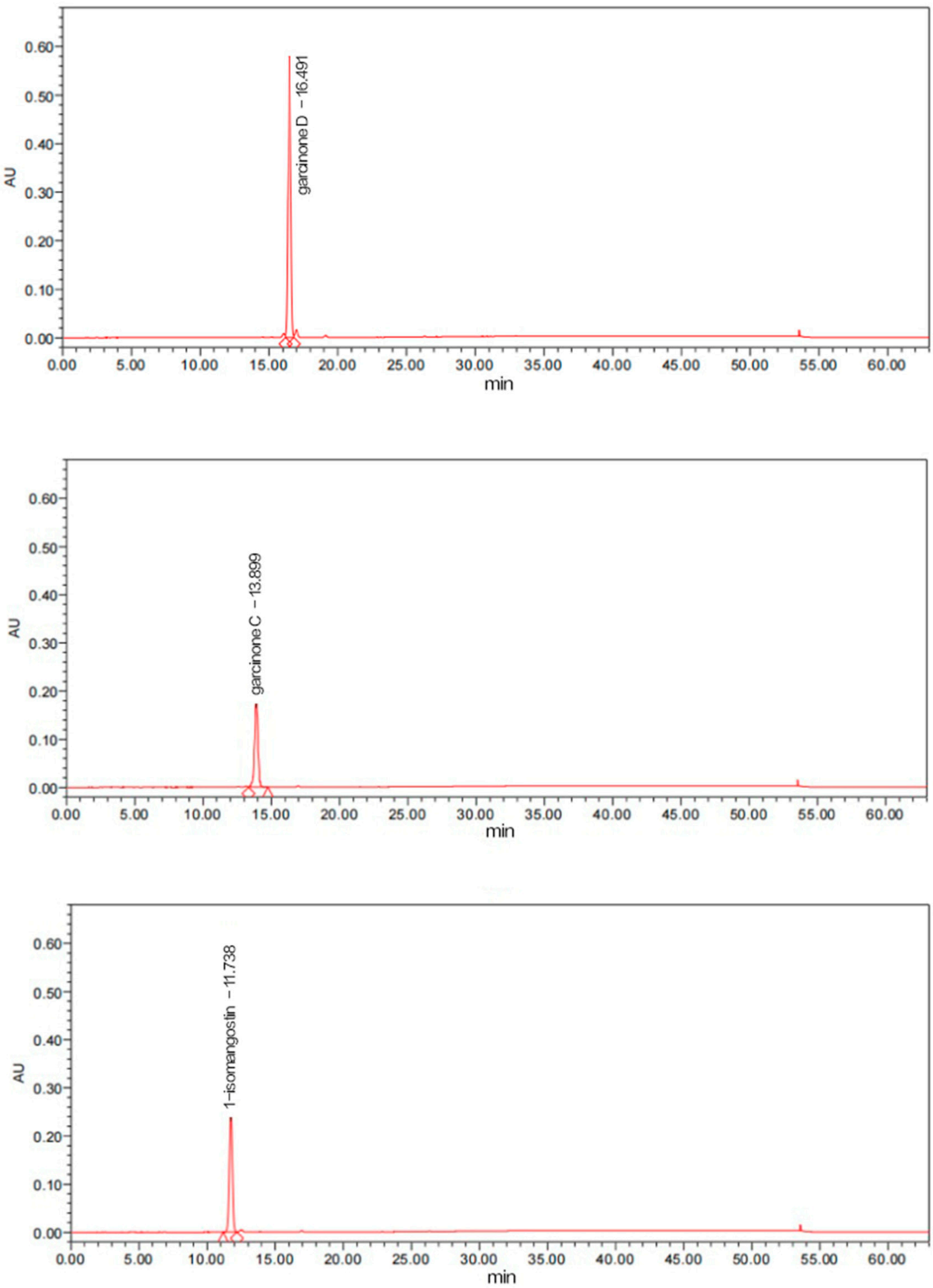

Figure S2: Chromatograms of xanthones
